# Temporal and spatial variation in bird and human use of beaches in southern California

**DOI:** 10.1186/2193-1801-2-38

**Published:** 2013-02-06

**Authors:** Kevin D Lafferty, Donald A Rodriguez, Angela Chapman

**Affiliations:** 1 Western Ecological Research Center, US Geological Survey c/o Marine Science Institute, University of California, Santa Barbara, CA 93106 USA; 2 Environmental Science and Research Management Program, California State University Channel Islands, Camarillo, CA 93012 USA; 3 Biology Program, California State University Channel Islands, Camarillo, CA 93012 USA

**Keywords:** Shorebirds, Beaches, Disturbance, Wrack

## Abstract

Southern California’s beaches can support a remarkable diversity of birds along the Pacific Flyway. We asked whether seasonal, annual, and spatial factors affect bird richness and abundance on public beaches. To do so, we conducted three years of monthly bird surveys on 12 sandy beaches in Ventura California. Across all surveys, we counted 22 shorebird species, 8 gull species, 24 other water bird species, and 24 landbird species. Sanderling, western gull, Heerman’s gull, willet, marbled godwit, and whimbrel were the most abundant members of the bird community. Beach wrack was uncommon, particularly where beaches were groomed, and did not have a large effect on bird abundance, though it was positively associated with overall bird richness. Beaches near estuaries tended to be wide, and such beaches had a higher richness and abundance of birds. Beaches with shallow slopes tended to have more gulls and shorebirds. People and (illegal) unleashed dogs were common, particularly at beaches fronted by houses. The abundance and richness of shorebirds and the richness of other waterbirds was lower where human activity was high. Bird richness and abundance was strongly affected by season, with the highest density of birds being seen during the fall shorebird migration. Gull abundance peaked earlier (August-September) than shorebird abundance (October through December). A brief pulse of shorebirds also occurred in May due to spring migration. Comparing these data with surveys in the 1990’s found no evidence for a decline in shorebirds over time, though black-bellied plover appear to still be recovering from the strong 1997-1998 ENSO. Opportunities to conserve birds on these beaches are limited, but could include enforcing leash laws and setting up human exclosures near estuary mouths.

## Introduction

Away from the volleyball courts and lifeguard stands, wildlife can be common at sandy beaches in southern California. Shorebirds forage on marine invertebrates in the swash zone and amongst the wrack line for insects and other intertidal arthropods. A few birds, such as least terns, snowy plovers, and killdeer sometimes nest. Birds that forage in the ocean, such as gulls and pelicans, use beaches to roost. Some are endangered and most are valued for their contribution to biodiversity. When considering how to plan for conservation of birds along recreational beaches, it is useful to know what factors drive their distributions. However, because these birds are highly mobile, and with the exception of the three nesting species above, non-territorial, their distributions can vary substantially in time and space. This variation can make it difficult to understand why birds can be common on one visit yet absent the next or why some beaches tend to support a high diversity of birds, whereas others possess only pigeons and gulls. Here, we attempt to explain patterns of bird abundance and richness on beaches in Ventura County, California, USA.

Foraging strategies affect bird distributions in time and space. Birds must feed frequently to maintain their high metabolisms, so they often congregate where food is plentiful (Hockey et al. 1992), and beaches vary in the food resources they contain. Crows, gulls, and pigeons are adept at foraging on human refuse and can benefit from litter where human activity is high. Some shorebirds feed on intertidal invertebrates, which are exposed at low tide. These birds shift their distributions according to the tides, feeding at low tide and roosting at high tide (Burger et al. 1977). Although beach topography, energy, and sand grain size can affect the type and abundance of prey (Evans and Dugan 1984), the beaches in our study area were relatively similar in this regard (Dugan et al. 2003). Beaches vary considerably in width and slope, and wider beaches should have more resources per linear kilometer. Shorebirds have also been shown to be more abundant on beaches with shallow slopes, presumably because these have more food resources (Neuman et al. 2008). Shorebirds feed on insects and amphipods associated with algal wrack deposited by waves on the upper beach (Dugan et al. 2003). If such birds seek out locations near off-shore sources of algae, seasonal and climatic variation in the distribution of wrack might affect temporal patterns in bird abundance (Revell et al. 2011). Vultures and gulls feed on stranded carcasses. But some municipalities groom recreational beaches in the summer, and even low-frequency grooming reduces invertebrate biomass and carcasses (Llewellyn and Shackley 1996; Dugan et al. 2000, 2003). Grooming also converts the vegetated upper strand habitat into un-vegetated dry sand zones (Dugan and Hubbard 2010).

Birds can use habitats in addition to the sandy beach. Therefore, the juxtaposition of habitats near the sandy beach can influence the bird community found there. Some species that use beaches for roosting forage primarily in estuaries (herons and egrets), along intertidal rocky shores (turnstones), or over ocean waters (pelicans, terns, cormorants). Landbirds (e.g., sparrows) occur on beaches, but they are dependent on vegetation for nesting and roosting. Raptors hunt near the beach if their roosts are near the shore, and peridomestic species like pigeons associate with parks that are often near beaches. Proximity to housing (increasing crows), proximity to estuary (increasing birds), and proximity to the rocky intertidal (increasing birds) affect the types of birds seen along a mix of sandy and rocky beach (Lafferty 2001). Other studies have also found that shorebirds are more common on beach transects near estuarine feeding areas (Colwell and Sundeen 2000; Neuman et al. 2008). In our study site, estuaries were the main type of adjacent habitat that varied in proximity from site to site.

Human activity, including dogs, horses, and vehicles, can disturb birds on beaches, and some species appear more sensitive to disturbance than others (Lafferty 2001; Glover et al. 2011). Humans and dogs are the main form of human activity at Southern California beaches (McCrary and Pierson 2000; Lafferty 2001). Unpredictable patterns of disturbance prevent birds from finding refuge, and while associations between instantaneous human and shorebird use might not be clear (McCrary and Pierson 2000; Lafferty 2001; Neuman et al. 2008), birds will move to areas protected from disturbance (Lafferty et al. 2006). Birds of prey predate on shorebirds and other species on beaches and may act as an additional source of disturbance for foraging and roosting birds. Therefore, the degree of disturbance may affect the spatial distribution of birds from beach to beach. In Ventura County, for example, shorebird abundance is consistently low on beaches with high average human use, presumably because disturbance causes birds to seek isolated locations (McCrary and Pierson 2000).

Bird abundance on a beach can change over short time intervals. In particular, birds respond to short-term variation in weather and tide (Ferns 1983). During storms, ocean-feeding birds, like pelicans, gulls and terns, halt foraging and roost on beaches. Low tides attract birds that forage in the intertidal zone (Neuman et al. 2008). Hubbard and Dugan (2003) found that low tides draw birds to forage in the intertidal zone in the spring, whereas in the fall, bird abundances are higher at high tide.

Studies of the distribution and abundance of shorebirds must consider seasonality, as this is often the factor that describes the greatest amount of variation in bird abundance and richness (Lafferty 2001; Hubbard and Dugan 2003). In southern California, peak migration occurs in the fall (beginning as early as July) as Arctic breeders pass through on their way to the southern hemisphere (Lafferty 2001; Hubbard and Dugan 2003). These birds pass through again during their return north in the spring, resulting in a small peak around April, followed by a minima in May and June (Lafferty 2001; Hubbard and Dugan 2003). Seasonal use varies by species; some birds are permanent residents, and many over winter. In our system, snowy plovers breed and winter on some beaches at our study site. Least terns, on the other hand, breed within the study area, but winter in the south.

Variation in climate can change the distribution of birds from one year to the next. In southern California, El Nino/Southern Oscillation (ENSO) conditions bring large storms and low productivity. This affects the prey base for birds and can alter characteristics of the physical habitat (Hubbard and Dugan 2003). Long-term trends in habitat destruction or restoration could lead to increasing variation over time. The world’s growing coastal population continues to rise (over 53% of the U.S. population lives in the coastal zone), increasing the encroachment of people into shorebird habitat (Burger and Gochfeld 1991). About half of the shorebird species in North America are in decline due to habitat destruction and degradation (Howe et al. 1989; Brown et al. 2001), and human disturbance (Davidson and Rothwell 1993).

We conducted 36 monthly surveys along twelve 1 km stretches of sandy beaches to help answer the following conservation related questions: 1) Are there monthly patterns in bird abundance and richness? 2) Are there significant differences from year to year? 3) How do site characteristics (abundance of algal wrack, human activity, or proximity to an estuary), drive spatial variation in bird communities? 4) Have certain shorebird species (black-bellied plover, snowy plover, willet, marbled godwit, whimbrel, sanderling) in 2007-2010 increased or declined compared to a similar study in 1994-1997?

## Materials and methods

The Ventura County coastline runs northwest to southeast along the Santa Barbara Channel of southern California and is about 62 km in length. Nearly all (93%) of the coast (Smith et al. 1976) consists of wave-swept (high energy) sandy intertidal beaches. Wetland habitats where shorebirds congregate are limited. The most extensive wetland in the county (1000 hectares) is on the Pt. Mugu Naval Weapons Station located along the central portion of the county coastline. Smaller amounts of estuarine habitat are also found at the Santa Clara River mouth and at ponds associated with various sewage treatment plants, electric generation plants, and agricultural runoff. Our sites were 12 1-km sandy beach segments that McCrary and Pierson (2002) randomly selected for shorebird surveys in 1994-1997. A map including these sites was published by Dugan et al. (2003). These beaches are subject to prevailing west winds and large winter swells from the west or northwest. Beaches in the central and southern part of Ventura County are also regularly exposed to southern swells in the summer months. Some of our study sites were groomed to various degrees. At 5^th^ Street, during 2007 and 2008, grooming occurred about every two months throughout the year (i.e., about 6 times/year) and in the period from 2009-2010, grooming was reduced to 4 times a year. Grooming efforts at Silver Strand focused on trash removal on the upper beach three times per week, but removing algae was not a priority. At Ormond 3, summer grooming occurred monthly on the northern most 200 meters of our transect, and winter grooming was occasional, following large storms which dump debris on the beach.

Observers identified and counted birds at these sites monthly from July 2007 to June 2010. Protocols were consistent with existing regulations in these areas (birds were not handled, or harassed), so no special permitting was required. The survey team consisted of one to four individual observers (at least one of which was specially trained in bird identification and survey protocols), taken from a pool of eight individuals. Trained observers accompanied student observers until they gained proficiency (5 survey experiences and lab identification). Observers walked 1 km transects recording shorebirds along the entire length. Transects were walked briskly (15-20 minutes) to avoid double counting. When possible, surveys were conducted during low incoming tides, but tidal height was used as a covariate (mean tidal height = 0.56 m, SD = 0.44). On the whole, surveys were done over two consecutive days each month (usually from north to south) to correspond with the tidal situation. Two survey teams were used to narrow the sampling window. However, data were not collected during poor weather, so some surveys were postponed until the next suitable day. Data collected included date, observer, start and stop time, approximate tidal height, weather and sea conditions, numbers of species, abundance, percentage of wrack cover, number of people, number of dogs (leashed or unleashed), and vehicles. Measures of average summer beach width and distance to the nearest estuary (in meters) were estimated from aerial photographs. The slope of the intertidal zone (in degrees) was taken from Dugan et al. (2003). Whereas McCrary and Pierson (2002) focused on six focal shorebird species, we counted all bird species on the beach.

### Statistical analyses

We used GLMs to analyze the effects of several variables (slope, amount of wrack, distance to estuary, human activity, tide, season, and year) on the abundance and species richness of four guilds of birds. There were no significant spatial autocorrelations or spatial trends in the dependent variables so we considered each site to be an independent observation. Still, we note that three sites: Ormond1, Ormond2, and Ormond3, were close together.

To reduce the statistical confounds of multiple comparisons, we did not analyze individual species responses, but divided the bird community into shorebirds, gulls, landbirds, and “other waterbirds”. Because birds of prey (osprey, Cooper’s hawk, kestrel) were seen on only four dates, they were not analyzed as an independent variable (preliminary analyses showed they did not negatively affect other bird species, and they were grouped with other landbirds). Species richness was considered an ordinal variable and bird abundance was considered a continuous variable.

Because observers had different abilities and biases, and more observers could lead to more complete counts, we checked for an effect of observer identity and observer number before proceeding with analyses. There was no observer effect on estimates of human activity or wrack. Both number of other waterbird species seen and gull abundance increased with the number of observers. Also, the abundance of shorebirds and other species varied by observer. Therefore, number of observers and observer identity were added to the statistical models to help control for this variation. However, to simplify the statistical results, we only report the main effects of interest (observer effects are listed in the statistical tables).

For all analyses, we considered month and year as categorical variables. To explain additional variation in bird richness and abundance, we also considered human activity (the number of humans plus dogs counted) and the amount of wrack (as a proportion of cover estimated visually on the beach). Although this measure of wrack was crude, this short cut was necessary to conduct transects rapidly so that counters did not flush or double count birds. Because we were primarily interested in wrack as a source of invertebrate food for birds, only wrack that included algae or marine plant material was included in our analyses (e.g., wrack consisting of sticks, trash and debris was not included). Because past studies indicated that the effect of tidal height varied by season (Hubbard and Dugan 2003), we included a tidal height by month interaction term. We considered three static habitat characteristics: summer beach width (to help account for habitat area), intertidal slope (and its interaction with tide), and the nearest distance from the transect to an estuary mouth (because many birds in our sample use both estuarine and shoreline habitats). Unfortunately, these site characteristics were highly correlated (with wider beaches close to estuaries). Subsequent analysis indicated that there was no positive effect of beach width on birds independent of distance to estuary. Therefore, we dropped beach width as a separate variable from the analysis, using estuary to represent both effects.

We also asked what factors explained human activity and wrack on the beach. As before, we considered month and year in the model. All beaches had public access with convenient public parking within walking distance. When analyzing human activity, we considered the amount of residential housing that fronted the beach as a static site variable. When analyzing factors affecting wrack, we considered the proportion of a transect likely to be groomed during a month of sampling (unfortunately, there was not sufficient information to accurately match grooming with particular surveys, given that Silver Strand weekly grooming did not focus on wrack, we discounted the grooming effort here to once a month).

To evaluate changes in birds among decades, we compared similar data on six focal shorebird species (black-bellied plover, snowy plover, willet, marbled godwit, whimbrel, sanderling) collected by McCrary and Pierson (2002) from the same sites between June 1994 and May 1997. In these analyses, we used month and site as a factor and also considered interactions between site and decade to determine if trends were similar at all sites. A problem with comparing our data with McCrary and Pierson (2002) is the lack of information on intervening years. To get an idea about intervening years from an independent data set, we plotted the data available for the six shorebird species from the Ventura Christmas Bird Count (CBC). The CBC conducts single-day surveys done in late December/Early January, usually along set routes, and often by the same observers. Ventura County CBC data were available for December 1997 - December 2010 (National Audubon Society 2011). These surveys were not matched to our sites so they were not directly compared to our data. Therefore, we simply asked if potential differences between our data and McCrary and Pierson (2002) were also reflected in the regional CBC. We also considered a longer time series for the Santa Barbara County CBC to further extend the generality of our findings.

Several of the variables had zero values and were over dispersed. We therefore used square-root transformations on bird abundance, distance to estuary, % wrack, and human activity. These transformations helped meet the assumptions of the general linear models of normally distributed residuals as indicated by normal quantile plots. To ease comparisons, we chose a consistent model structure for all analyses. Therefore, all variables were included in the final models whether significant or not (all were significant in some of the analyses). With the exception of tide by month and tide by slope, first-order interactions were removed due to overall insignificance. Analyses of richness were done with ordinal logistic regression. All other analyses were standard least squares. Post hoc tests using Tukey HSD were done for month and year to determine groupings in the temporal data. To simplify presentation, we present the analyses in tabular form, indicating the sign of the effect and the level of statistical significance for each independent factor. To ease comparison and consistency with the least-squares statistical tables, the ordinal regressions report slope coefficients as positive if the independent variable was associated with an increase in species richness.

## Results

**Figure 1:**
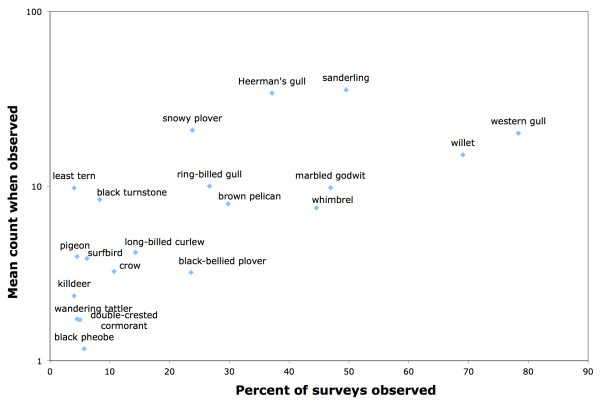
**Frequency and abundance of the 20 most frequently seen bird species on Ventura county beaches.** Average abundance is the product of % seen and mean count when seen. There were 58 other species seen in our survey that were seen on less than 4% of surveys.

We identified 78 bird species, though ten of these species were only seen once. On average, there were 5.6 (+/- 3.2) species per km surveyed. Western gull (78%), willet (69%), sanderling (50%), marbled godwit (47%), whimbrel (45%), and Heerman’s gull (37%) were present on more than one third of the surveys (Figure 1). The main drivers of overall bird richness were month, algal wrack, and human activity (Table 1).

**Table 1:** **Ordinal linear model of bird richness**

Independent variable	Effect	df	Chi-Square	P
Slope	−0.09	1	2.56	0.1098
Month		11	38.14	<.0001
Year		3	2.30	0.5116
Sqrt(% algal wrack)	0.20	1	8.72	0.0031
Sqrt(Distance to estuary)	−0.01	1	3.27	0.0705
Sqrt(Human activity)	−0.11	1	7.82	0.0052
Tide	−0.01	1	0.01	0.9138
Month*Tide		11	18.23	0.0763
Tide*Slope	−0.05	1	2.15	0.143
# observers		1	0.36	0.5458
G		1	0.01	0.9155
W		1	0.52	0.4702
F		1	0.02	0.8859
K		1	0.09	0.7697
T		1	0.00	0.9754
N		1	1.00	0.317
C		1	5.31	0.0212
B		1	0.24	0.6275

R-square = 0.09, ChiSquare = 192.2 DF = 40, P <.0001. Estuary refers to the inverse of the shortest distance from the transect to an estuary. Letter codes refer to observers. Effect sign is negative to that given in statistical output to indicate the effect of the independent variable on the dependent variable as in a General Linear Model.

On average there were 87.9 (+/-167) birds per km. There were 22 species of shorebirds, which represented 49% of the birds counted. Of the 44.4 (+/- 82.4) shorebirds per survey, sanderling (40%), willet (23%), snowy plover (11%), and marbled godwit (10%) were the most abundant. There were eight species of gulls and the 33.4 (+/- 75.6) gulls per survey comprised 38% of the birds. The most abundant were western gull (47%), Heerman’s gull (38%), ring-billed gull (8%), and mew gull (7%). There were 24 other waterbird species, but these amounted to only 10% of the birds counted (9.1 (+/- 98.3) other waterbirds per survey). The most common species in this category were western/Clarke’s grebe (55%), brown pelican (24%), royal tern (6%), and least tern (5%). Landbirds were the least common group (1% of the total, 1.0 (+/- 2.7) landbirds per transect), and were represented by 24 species, of which the American crow (34%), rock pigeon (19%), house finch (15%), and turkey vulture (8%) were the most common. The main drivers of overall bird abundance were the distance to the nearest estuary (or beach width), month, and beach slope (Table 2). A full list of the less common species is available as a report from the second author (Rodriguez et al. 2011).

**Table 2:** **General linear model of Sqrt(bird abundance)**

Independent variable	Effect	df	SS	F Ratio	P
Intercept	8.34				<.0001
Slope	−0.43	1	179.45	9.19	0.0026
Month		11	639.89	2.98	0.0008
Year		3	118.76	2.03	0.1096
Sqrt(% algal wrack)	0.11	1	9.36	0.48	0.4892
Sqrt(Distance to estuary)	−0.03	1	382.38	19.58	<.0001
Sqrt(Human activity)	−0.14	1	51.75	2.65	0.1044
Tide	0.04	1	0.53	0.03	0.8688
Month*Tide		11	661.81	3.08	0.0006
Tide*Slope	−0.05	1	6.00	0.31	0.5796
# observers		1	48.02	2.46	0.1177
G		1	97.35	4.98	0.0262
W		1	87.45	4.48	0.035
F		1	59.67	3.06	0.0813
K		1	96.38	4.94	0.0269
T		1	75.45	3.86	0.0501
N		1	19.06	0.98	0.3238
C		1	9.27	0.47	0.4913
B		1	0.24	0.01	0.9111

R-square = 0.39, F40,379 = 6.06, P <.0001. Estuary refers to the inverse of the shortest distance from the transect to an estuary. Letter codes refer to observers.

Beaches with flatter slopes tended to have more shorebirds and gulls. Specifically, as for past studies (Neuman et al. 2008), the abundance of shorebirds decreased with beach slope (Table 3, Table 4). Furthermore, focal shorebird richness (Table 5), but not overall shorebird richness (Table 6), declined with beach slope. Like shorebirds, the richness (Table 7) and abundance (Table 8) of gulls decreased with the slope of the beach. However, the richness of other waterbirds increased with beach slope (Table 9).

**Table 3:** **General linear model of Sqrt(shorebird abundance)**

Independent variable	Effect	df	SS	F Ratio	P
Intercept	5.73				<.0001
Slope	−0.25	1	60.26	5.72	0.0173
Month		11	582.50	5.02	<.0001
Year		3	8.61	0.27	0.8456
Sqrt(% algal wrack)	0.09	1	6.37	0.60	0.4373
Sqrt(Distance to estuary)	−0.02	1	150.26	14.25	0.0002
Sqrt(Human activity)	−0.12	1	36.50	3.46	0.0636
Tide	−0.16	1	7.63	0.72	0.3956
Month*Tide		11	608.89	5.25	<.0001
Tide*Slope	−0.02	1	0.98	0.09	0.7604
# observers		1	26.81	2.54	0.1117
G		1	57.48	5.45	0.0201
W		1	69.97	6.64	0.0104
F		1	42.14	4.00	0.0463
K		1	37.06	3.51	0.0616
T		1	41.21	3.91	0.0488
N		1	8.44	0.80	0.3717
C		1	18.20	1.73	0.1898
B		1	0.25	0.02	0.8788

R-square = 0.41, F40,379 = 6.71, P <.0001. Estuary refers to the inverse of the shortest distance from the transect to an estuary. Letter codes refer to observers.

**Table 4:** **General linear model of Sqrt(focal shorebird abundance)**

Independent variable	Effect	df	SS	F Ratio	P
Intercept	5.49				<.0001
Slope	−0.24	1	56.34	5.50	0.0196
Month		11	612.89	5.44	<.0001
Year		3	10.67	0.35	0.7914
Sqrt(% algal wrack)	0.04	1	1.41	0.14	0.7109
Sqrt(Distance to estuary)	−0.02	1	119.55	11.67	0.0007
Sqrt(Human activity)	−0.12	1	42.02	4.10	0.0436
Tide	−0.11	1	3.94	0.38	0.5355
Month*Tide		11	636.98	5.65	<.0001
Tide*Slope	−0.02	1	0.74	0.07	0.7879
# observers		1	45.30	4.42	0.0362
G		1	84.15	8.21	0.0044
W		1	97.72	9.54	0.0022
F		1	56.43	5.51	0.0195
K		1	50.72	4.95	0.0267
T		1	57.68	5.63	0.0182
N		1	16.28	1.59	0.2083
C		1	30.82	3.01	0.0837
B		1	0.02	0.00	0.965

R-square = 0.41, F40,379 = 6.84, P <.0001. Estuary refers to the inverse of the shortest distance from the transect to an estuary. Letter codes refer to observers.

**Table 5:** **Ordinal linear model of focal shorebird richness**

Independent variable	Effect	df	Chi-Square	P
Slope	−0.15	1	6.24	0.0125
Month		11	100.13	<.0001
Year		3	1.52	0.6786
Sqrt(% algal wrack)	0.01	1	0.02	0.8751
Sqrt(Distance to estuary)	0.00	1	0.66	0.416
Sqrt(Human activity)	−0.14	1	11.19	0.0008
Tide	−0.15	1	2.07	0.1504
Month*Tide		11	8.75	0.6451
Tide*Slope	−0.06	1	3.27	0.0705
# observers		1	0.90	0.3417
G		1	2.69	0.1008
W		1	1.63	0.2019
F		1	2.30	0.1295
K		1	0.57	0.4514
T		1	0.61	0.4339
N		1	0.14	0.7109
C		1	0.19	0.6597
B		1	0.78	0.3759

R-square = 0.15, ChiSquare = 226.5 DF = 40, P <.0001. Estuary refers to the inverse of the shortest distance from the transect to an estuary. Letter codes refer to observers. Effect sign is negative to that given in statistical output to indicate the effect of the independent variable on the dependent variable as in a General Linear Model.

**Table 6:** **Ordinal linear model of shorebird richness**

Independent variable	Effect	df	Chi-Square	P
Slope	−0.09	1	2.50	0.1138
Month		11	77.34	<.0001
Year		3	1.50	0.6831
Sqrt(% algal wrack)	0.08	1	1.15	0.2831
Sqrt(Distance to estuary)	0.00	1	1.39	0.2376
Sqrt(Human activity)	−0.14	1	12.01	0.0005
Tide	−0.08	1	0.58	0.4474
Month*Tide		11	13.41	0.2676
Tide*Slope	−0.04	1	1.49	0.2227
# observers		1	0.91	0.3404
G		1	1.87	0.1712
W		1	1.31	0.2525
F		1	2.25	0.1337
K		1	0.99	0.3196
T		1	0.97	0.3245
N		1	0.18	0.6689
C		1	0.12	0.7275
B		1	1.12	0.2904

R-square = 0.11, ChiSquare = 198.1 DF = 40, P <.0001. Estuary refers to the inverse of the shortest distance from the transect to an estuary. Letter codes refer to observers. Effect sign is negative to that given in statistical output to indicate the effect of the independent variable on the dependent variable as in a General Linear Model.

**Table 7:** **Ordinal linear model of gull richness**

Independent variable	Effect	df	Chi-Square	P
Slope	−0.13	1	4.66	0.0309
Month		11	40.95	<.0001
Year		3	4.97	0.1742
Sqrt(% algal wrack)	0.08	1	1.35	0.2447
Sqrt(Distance to estuary)	−0.01	1	2.69	0.1007
Sqrt(Human activity)	0.00	1	0.01	0.9096
Tide	−0.04	1	0.16	0.689
Month*Tide		11	20.03	0.0449
Tide*Slope	−0.05	1	2.07	0.1504
# observers		1	0.49	0.4818
G		1	0.00	0.9869
W		1	0.80	0.371
F		1	0.15	0.7025
K		1	0.11	0.7385
T		1	0.09	0.764
N		1	0.47	0.4908
C		1	4.89	0.027
B		1	1.48	0.2244

R-square = 0.13, ChiSquare = 162.0 DF = 40, P <.0001. Estuary refers to the inverse of the shortest distance from the transect to an estuary. Letter codes refer to observers. Effect sign is negative to that given in statistical output to indicate the effect of the independent variable on the dependent variable as in a General Linear Model.

**Table 8:** **General linear model of Sqrt(gull abundance)**

Independent variable	Effect	df	SS	F Ratio	P
Intercept	5.01				<.0001
Slope	−0.27	1	70.05	5.13	0.024
Month		11	602.81	4.02	<.0001
Year		3	93.69	2.29	0.078
Sqrt(% algal wrack)	0.01	1	0.11	0.01	0.9292
Sqrt(Distance to estuary)	−0.02	1	216.49	15.87	<.0001
Sqrt(Human activity)	−0.03	1	1.95	0.14	0.7052
Tide	0.22	1	15.20	1.11	0.2919
Month*Tide		11	344.42	2.30	0.01
Tide*Slope	−0.04	1	4.91	0.36	0.5491
# observers		1	37.58	2.75	0.0978
G		1	57.84	4.24	0.0402
W		1	28.38	2.08	0.15
F		1	19.63	1.44	0.2311
K		1	46.63	3.42	0.0653
T		1	36.09	2.65	0.1047
N		1	18.78	1.38	0.2414
C		1	1.23	0.09	0.7644
B		1	0.86	0.06	0.8015

R-square = 0.29, F40,379 = 4.00, P <.0001. Estuary refers to the inverse of the shortest distance from the transect to an estuary. Letter codes refer to observers.

**Table 9:** **Ordinal linear model of other waterbird richness**

Independent variable	Effect	df	Chi-Square	P
Slope	0.16	1	4.33	0.0375
Month		11	16.12	0.1366
Year		3	19.28	0.0002
Sqrt(% algal wrack)	0.29	1	12.11	0.0005
Sqrt(Distance to estuary)	−0.02	1	21.98	<.0001
Sqrt(Human activity)	−0.12	1	8.10	0.0044
Tide	0.21	1	1.92	0.1657
Month*Tide		11	17.25	0.1007
Tide*Slope	0.06	1	1.81	0.1783
# observers		1	5.65	0.0175
G		1	4.19	0.0406
W		1	9.61	0.0019
F		1	3.50	0.0614
K		1	0.80	0.3714
T		1	9.68	0.0019
N		1	7.98	0.0047
C		1	16.90	<.0001
B		1	0.05	0.825

R-square = 0.26, ChiSquare = 238.2 DF = 40, P <.0001. Estuary refers to the inverse of the shortest distance from the transect to an estuary. Letter codes refer to observers. Effect sign is negative to that given in statistical output to indicate the effect of the independent variable on the dependent variable as in a General Linear Model.

Wrack appeared to positively affect only a few aspects of the bird community. Overall, wrack with algae was variable, but relatively uncommon on Ventura beaches, amounting to 6.7% (+/- 9.1 S.D.) cover. Not surprisingly, the frequency of grooming (which was positively associated with housing development, P < 0.0001) reduced wrack cover; there was also a positive association between wrack and tidal height (Table 10). 2009 had more wrack than the other years and wrack was more abundant July-January than in the rest of the year, with the exception of a relatively high abundance of wrack during low tide in April. The richness of other waterbirds (Table 9) was positively associated with the proportion of beach covered with algal-associated wrack. For example, the statistical model suggested that one additional species of bird was expected to be present if wrack cover increased from 0 to 26%. However, the richness of gulls (Table 7), shorebirds (Table 6), and landbirds (Table 11), were not associated with variation in wrack cover.

**Table 10:** **General linear model Sqrt(% algal wrack)**

Independent variable	Effect	df	SS	F Ratio	P
Month		11	77.8	3.12	0.0005
Year		3	90.2	13.3	<.0001
Grooming		1	32.6	3.6	0.007
Tide	0.20	1	16.6	7.3	0.007
Tide*Month		11	42.9	1.7	.0672

R-square = 0.29, F30,389 = 5.0, P <.0001.

**Table 11:** **Ordinal linear model of landbird richness**

Independent variable	Effect	df	Chi-Square	P
Slope	0.06	1	0.52	0.4729
Month		11	21.42	0.0293
Year		3	10.51	0.0147
Sqrt(% algal wrack)	0.16	1	3.02	0.0825
Sqrt(Distance to estuary)	0.01	1	11.73	0.0006
Sqrt(Human activity)	0.03	1	0.43	0.5104
Tide	0.06	1	0.16	0.6937
Month*Tide		11	8.52	0.6664
Tide*Slope	−0.10	1	5.15	0.0232
# observers		1	1.07	0.3
G		1	1.56	0.2114
W		1	0.28	0.5955
F		1	0.91	0.3397
K		1	0.18	0.6688
T		1	1.26	0.2626
N		1	0.26	0.6073
C		1	2.17	0.1405
B		1	0.08	0.7723

R-square = 0.13, ChiSquare = 89.27 DF = 40, P <.0001. Estuary refers to the inverse of the shortest distance from the transect to an estuary. Letter codes refer to observers. Effect sign is negative to that given in statistical output to indicate the effect of the independent variable on the dependent variable as in a General Linear Model.

The presence of an estuary (or its correlation with beach width) affected some measures of birds. Most beaches were a kilometer or more from an estuary (one was 14 km from the nearest estuary), but Ormond Beach 3, Ormond Beach 2 and Surfer’s Knoll were within 100 m of an estuary mouth. The abundance (Table 8, Table 3, Table 12) of birds was positively associated with proximity to an estuary mouth, with the exception that the abundance (Table 13) and richness (Table 11) of land birds increased with the distance from an estuary. Whether these effects were due to the estuary itself or the tendency for beaches near estuaries to be wider was not possible to determine from our data.

**Table 12:** **General linear model of Sqrt(other waterbird abundance)**

Independent variable	Effect	df	SS	F Ratio	P
Intercept	0.50				0.4455
Slope	0.01	1	0.06	0.01	0.9251
Month		11	34.58	0.47	0.9227
Year		3	25.53	1.27	0.2859
Sqrt(% algal wrack)	0.11	1	9.26	1.38	0.2414
Sqrt(Distance to estuary)	−0.01	1	34.78	5.17	0.0235
Sqrt(Human activity)	−0.10	1	24.42	3.63	0.0574
Tide	0.19	1	11.15	1.66	0.1985
Month*Tide		11	65.18	0.88	0.5588
Tide*Slope	−0.01	1	0.64	0.10	0.7577
# observers		1	4.37	0.65	0.4205
G		1	1.11	0.17	0.6847
W		1	4.08	0.61	0.4362
F		1	0.00	0.00	0.9786
K		1	0.06	0.01	0.9267
T		1	1.80	0.27	0.6048
N		1	7.50	1.12	0.2916
C		1	12.46	1.85	0.1743
B		1	0.08	0.01	0.9121

R-square = 0.21, F40,379 = 2.58, P <.0001. Estuary refers to the inverse of the shortest distance from the transect to an estuary. Letter codes refer to observers.

**Table 13:** **General linear model of Sqrt(landbird abundance)**

Independent variable	Effect	df	SS	F Ratio	P
Intercept	−0.41				0.0519
Slope	−0.01	1	0.20	0.28	0.5981
Month		11	14.27	1.83	0.0480
Year		3	7.26	3.41	0.0177
Sqrt(% algal wrack)	0.04	1	1.32	1.86	0.1732
Sqrt(Distance to estuary)	0.00	1	4.35	6.13	0.0137
Sqrt(Human activity)	0.02	1	0.98	1.39	0.2399
Tide	0.06	1	1.14	1.61	0.2058
Month*Tide		11	5.91	0.76	0.6840
Tide*Slope	−0.02	1	0.82	1.16	0.2831
# observers		1	0.23	0.32	0.5726
G		1	0.14	0.19	0.6623
W		1	0.12	0.16	0.6855
F		1	0.16	0.23	0.6338
K		1	0.57	0.80	0.3723
T		1	1.32	1.85	0.1741
N		1	0.37	0.52	0.4702
C		1	0.79	1.12	0.2915
B		1	0.01	0.01	0.9285

R-square = 0.16, F40,379 = 1.86, P 0.0016. Estuary refers to the inverse of the shortest distance from the transect to an estuary. Letter codes refer to observers.

On average, there were 16.2 people (57.7 S.D.), and 1.6 dogs (57.7 S.D.) (81% unleashed, despite posted leash laws) per km of beach. Silverstrand had the most human activity (88 people and 2.4 dogs per km), whereas Ormond 2 had the least (2.1 people and 0.1 dogs per km). Human activity was positively associated with the proportion of the beach that was fronted by housing development and varied by month, with most activity occurring during August and the least in May (Table 4). Human activity appeared to negatively affect some types of birds on beaches. The richness of shorebirds (Table 6) and other waterbirds (Table 9) was negatively associated with short-term effects of human activity, as measured by the sum of humans and dogs counts. The abundance of shorebirds (Table 3) and other waterbirds (Table 12) was marginally negatively associated with human activity. However, neither measure of gulls (Table 8, Table 7) nor of land birds (Table 13, Table 11) was associated with human activity. The statistical models estimated that one bird species (Table 1) was displaced by nine humans (or dogs) per kilometer. Repeating the analysis of McCrary and Pierson (2000) for our data, but focusing on their focal shorebird species, there were negative associations between the average amount of human activity on a beach and the average shorebird abundance (Table 4) and richness (Table 5) on a beach.

There was not a consistent effect of tidal height on birds. Specifically, tidal height was not a significant factor on its own, but there was sometimes a significant interaction with month, indicating that the effect of tide, when it occurred, was inconsistent from season to season (see all Tables). Tidal height did not appear to affect bird richness. Shorebird abundance (Table 3) increased with tidal height in October, but decreased with tidal height in May. Gull abundance (Table 8) also increased with tidal height in October, but had a negative association with tidal height in August. The abundance of other waterbirds (Table 12) or landbirds (Table 13) was not significantly associated with tidal height during any month.

**Figure 2:**
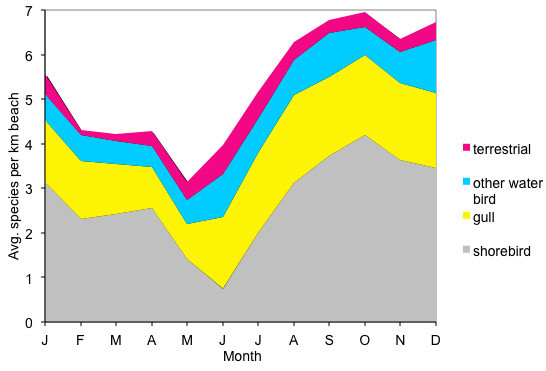
**Average monthly species richness of birds at Ventura County beaches, divided into terrestrial birds, shorebirds, gulls, and other waterbirds.** The highest richness is driven by fall migration of shorebirds.

**Figure 3:**
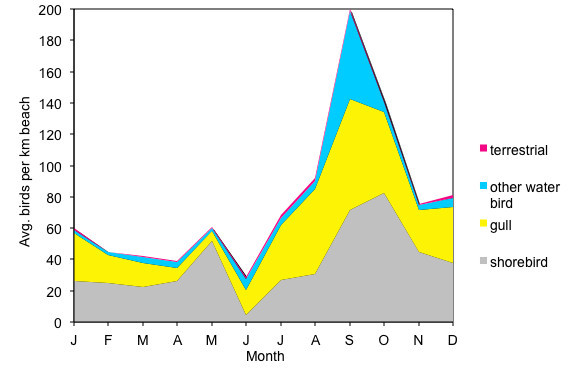
**Average monthly density of birds at Ventura County beaches, divided into terrestrial birds, shorebirds, gulls, and other waterbirds.** The highest density is driven by fall migration of shorebirds. A second peak can be seen for spring shorebird migration.

**Figure 4:**
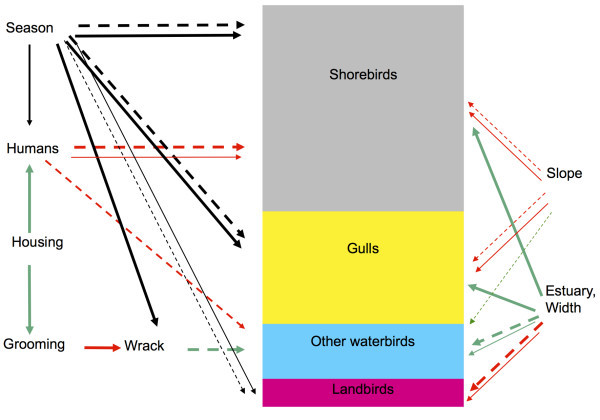
**The direction and strength of associations among variables, partitioned among four categories of birds on the beach.** Solid arrows indicate associations with abundance, whereas dashed arrows indicate associations with species richness. Red arrows are negative associations, green arrows are positive associations and black arrows are categorical effects. Arrow width indicates the number of significant digits in the P value. This is not the same as the slope of the effect or the strength of a standardized regression coefficient, so the figure should not be interpreted as a path diagram or structural equation model. “Housing” refers to residential development.

As is well known for migratory species, many birds showed seasonal variation. Seasonality is arguably best measured on a species by species basis, but was clear in the monthly data for some categories of birds. The abundance and richness of gulls (Table 8, Table 7), shorebirds (Table 3, Table 6), and landbirds (Table 13, Table 11), changed significantly from month to month. The total richness of birds was lowest in February, March, May, and June and highest in October through December (Figure 2). The total abundance of birds was less than average in February, April, and June and more than average in August and September (Figure 3). However, these patterns differed by bird category. Shorebirds were least diverse in May and June and most diverse in October through December (Figure 2). Despite low richness, shorebirds were abundant in May, during spring migration (Figure 3). Shorebirds were most abundant in October, during fall migration (Figure 3). In contrast, gulls were less abundant in April and May and more abundant in August and September (Figure 3). The richness of gulls was highest in the summer and fall and lowest in the winter and spring (Figure 2). Landbirds were less diverse in February and March and more diverse in June and July (Figure 2). As a group, other waterbirds did not show seasonal patterns in richness or abundance, though seasonal trends in individual species within this group are well known. Figure 4 shows the hypothesized interactions among these effects.

Although individual species varied considerably from year to year, there were only a few differences from 2007 to 2010 in our broader categories of birds. Specifically, the year 2007 had a lower than average richness for other waterbirds (Table 9) and in 2009, there was a higher than average abundance and richness of landbirds (Table 13, Table 11). Our data for the six focal shorebird species monitored by McCrary and Pierson (2000) showed some decadal changes (1994-1997 vs. 2007-2011). Site by decade interactions were common, indicating that some sites saw increases in abundance across decades but others saw decreases. Of the individual species, willet (P = 0.0035) and black-bellied plover (P = 0.0001) decreased in abundance, but godwit (P = 0.01), whimbrel (P = 0.0005), and snowy plover (0.0008) increased in abundance. Sanderling did not change significantly between decades. Overall, for the total abundance of the six focal species of shorebirds, there was no effect of decade on total abundance. However, there was a significant trend toward slightly lower shorebird richness in the latter decade (2.9 vs. 2.5 out of 6 species, on average, P = 0.0002). In contrast, the more regular surveys of the Christmas Bird Count data for Ventura County showed an increase in all six shorebird species between December 1997 and December 2009 (average slope of birds per unit effort vs. year = +0.07, SD = 0.05 (National Audubon Society 2011). Our analysis of the longer term CBC counts from adjacent Santa Barbara County, California, showed slight long-term positive trends for all species but black-bellied plover (for 1990-2009, average slope of birds per unit effort vs. year = 0.02, SD = 0.03). However, this time series showed a steep drop in the counts of all six species coincident with the 1997-1998 ENSO event (for 1997-1998, average slope of birds per unit effort vs. year = -0.32, SD = 0.19. Since then, all species but black-bellied plovers have recovered to pre-ENSO levels (for 1998-2009, average slope of birds per unit effort vs. year = 0.04, SD = 0.06 (National Audubon Society 2011). In sum, these results suggest that there has not been a long-term decline in birds, except for a lingering effect of the 1997-1998 ENSO on black-bellied plovers.

## Discussion

Our study provides basic information on the community of birds that uses beach habitat in Ventura County California. The richness, density, and seasonality of the bird community found along Ventura County beaches was similar to a comparable year-round study in neighboring Santa Barbara County conducted in 1999 (Lafferty 2001). A key difference was that, in Santa Barbara, winter bird abundances were as high as fall bird abundances (whereas winter counts were half of fall abundances in Ventura). Not surprisingly, there were differences between the two studies in the relative abundance of some of the bird species. For instance, among the common birds, American crow, semipalmated plover, western sandpiper, black-bellied plover, and western snowy plover were more abundant in Santa Barbara whereas brown pelican was more abundant in Ventura.

Birds have been recovering from a large ENSO a decade before our surveys (Hubbard and Dugan 2003), but there was no evidence for a long-term decline in bird abundance or richness. Site characteristics also affected the bird community, with wide beaches close to estuaries supporting more waterbirds. Algal wrack was positively associated with the abundance of other waterbirds, but not shorebirds. Development was negatively associated with birds, presumably because housing was associated with human activity, which negatively affected the abundance and richness of shorebirds and other waterbirds. Figure 4 summarizes these hypothesized causal effects.

Unfortunately, more birds were seen when there were more observers and some observers reported more species and birds than others, presumably due to variation in observer experience that our training did not compensate for. Although observer effects were detected and controlled for statistically, including observer as an effect did not change the qualitative nature of the results. Ideally, bird surveys should standardize observer number and experience, though this can be difficult for multi-site projects such as ours.

It seems surprising that wrack did not have a strong effect on birds, but this is consistent with other studies. Dugan et al. (2003) found no association between winter shorebird richness or abundance with the mean cover of wrack on 15 southern California beaches, even though invertebrate prey availability was directly associated with wrack; only the abundance of snowy plovers and black-bellied plovers was associated with the amount of wrack on a beach. This previous study, though it measured wrack rigorously over two-weeks in the fall, did not match wrack and bird data from the same surveys, potentially explaining why our approach was able to detect broader associations with wrack both within and among sites. Still, the associations we did see with wrack were limited and inconsistent, either because birds do not aggregate where there is wrack, or because our measures of wrack were too coarse, or because wrack was relatively rare on these beaches. In comparison, the abundance of wrack seems more strongly associated with the richness of shorebirds at Santa Barbara and San Luis Obispo County sites where wrack is more abundant overall (J. Dugan pers. Comm.).

It is not surprising that birds were more abundant and diverse on wide, flat, sandy beaches near the mouths of estuaries. This is consistent with studies showing that bird abundance and richness increases near an estuary mouth (Lafferty 2001; Neuman et al. 2008), studies showing that birds are less common on narrow, armored beaches (Dugan and Hubbard 2006; Dugan et al. 2008), and studies showing that shorebirds are more abundant at beaches with shallow-sloping intertidal zones (Neuman et al. 2008). Estuaries are a source of sand, and this expands the habitat available for birds. Greater beach area can also reduce the density of bird-human interactions. In addition, many birds that use beaches also forage or roost in estuary habitat. Estuary habitat in southern California has been lost and degraded as estuaries and adjacent lands in Ventura County have been developed as harbors, sources of sewage treatment, sand mining, industrial development, and parking lots. These impacts preceded our study and protections in the last several decades have slowed and sometimes reversed losses.

A variable effect of tide on birds, depending on the season, was qualitatively consistent with past studies in this region. Hubbard and Dugan (2003) found that shorebirds were more common at low tides in the spring. This was expected because low tide exposes more beach habitat and increases foraging opportunities. However, Hubbard and Dugan (2003) were not able to explain why shorebirds were more common at high tide in the winter and fall (this could be related to confounds between time of day and tidal height and season). In our survey, shorebirds were also more common at high tide from September to December but otherwise not affected by tide or more common at low tide. We also do not know why birds would be more abundant at high tide. One possibility is that high tides in the fall cause beach hoppers to seek higher elevations, which exposes them to predation by shorebirds.

As expected, bird migrations drove dramatic changes in the abundance and richness of shorebirds and gulls. Other waterbirds did not show a strong seasonal pattern. Fall migration was associated with a large and sustained increase in bird abundance and richness. In contrast, spring migration was brief and moderate in scope. Birds were uncommon in May and June. Local breeders (mostly snowy plovers and least terns) were limited to a few sites with active protection efforts.

Weather and ocean conditions vary from year to year and this could have explained why some of our results showed annual differences. Still, most patterns were consistent from year to year. Decadal differences between our data and the data of McCrary and Pierson (2002) were mixed for their six focal shorebird species. In contrast, Ventura Christmas Bird Counts (CBCs) show consistent increases in counts for the period after McCrary and Pierson’s (2002) study through the last year of our surveys, indicating that declines in willet and black-bellied plover have not been gradual over this time. One possible explanation is the strong ENSO in the winter of 1997-1998 led to a decline and eventual recovery of shorebirds. This hypothesis cannot be evaluated in Ventura county because the Ventura CBC data started after the ENSO and the data of McCrary and Pierson (2002) predate the ENSO. However, in neighboring Santa Barbara County, the CBC extends farther back in time and indicates that the ENSO was associated with sharp drops in the six focal shorebird species. Trends in these shorebirds over time have been positive since the 1997-1998 El Nino in both the Santa Barbara and Ventura CBCs. So, although snowy plover, marbled godwit, and whimbrel eventually exceeded the immediate pre-El Nino period sampled by McCrary and Pierson (2002), willet and black-bellied plover have been slower to recover. Although an ENSO effect seems plausible, we cannot, from the available data, determine if there have been any additional human-induced changes in shorebirds over the last decade in Ventura County.

Although we did not measure human disturbance directly, other studies have found that birds are disturbed several times per hour on recreational beaches in southern California (Lafferty 2001). Studies that have failed to find real-time negative associations between birds and human activity on beaches have assumed that disturbances are too random in time and space for birds to adjust to (McCrary and Pierson 2000; Lafferty 2001; Yasue 2006; Neuman et al. 2008). Our data are consistent with McCrary and Pierson’s (2000) interpretation that long-term average human use leads to broad-scale, but difficult to detect, changes in average bird distributions among beaches, so that sites with many humans have low richness and abundance of shorebirds. Similarly, Lafferty et al. (2006) found that birds moved into a small area where human disturbance was excluded. We did not separate effects of dogs and humans, but birds are known to respond to dogs at greater distances than humans, perhaps because birds instinctively view dogs as predators (Lafferty 2001). Most of the beaches in our study receive substantial human use and this will only increase with time due to continued growth of coastal populations in southern California (population along the Pacific Coast has increased from 47 million in 1960 to 87 million in 2008). Some management actions might reduce disturbance to birds on beaches. One of the most obvious options is to leash pets on beaches as some dogs will actively chase birds (Lafferty 2001). California law specifically prohibits off-leash pets on beaches, but these laws are not commonly posted or enforced. Even when posted, dog owners often ignore the laws because local law enforcement agencies consider leashing a low priority. Another management action that can reduce disturbance to birds on beaches is to rope off areas where birds concentrate, such as near the mouths of estuaries (Ikuta and Blumstein 2003; Lafferty et al. 2006). Surveys of beach users in Australia indicate that most beach users support protecting birds on beaches so long as walking is not prohibited along the wet sand (Glover et al. 2011).

Special management actions for western snowy plovers and California least terns are conducted within the portions of California State Seashores that are owned by the California Department of Parks and Recreation. An example is the Point Mugu State Seashore (consists of lands extending from Ormond Beach, Point Mugu State Beach, and Leo Carillo State Beach all within the study area), California Department of Parks and Recreation has conducted management activities since 1991. Strategies include interpretation, enforcement, monitoring, predator trapping, nest exclosures, and symbolic fences. Interpretative efforts include signage at nesting areas, brochures, small handout cards with photographs and information on western snowy plovers, and public outreach programs (e.g., presentations and field trips).

In summary, Ventura County beaches support a diverse and abundant community of birds. In addition to shorebirds, these include many gulls, other waterbirds, and a few landbirds. Many birds have seasonal migrations, resulting in substantial month-to-month variation in abundance and richness. Humans affect birds directly through disturbance and indirectly through the practice of beach grooming. As a group, shorebirds have not suffered human-associated declines in the last decade, which is comforting considering the massive amount of habitat loss this group experienced in prior decades.
